# Giant sequoia (*Sequoiadendron giganteum*) in the UK: carbon storage potential and growth rates

**DOI:** 10.1098/rsos.230603

**Published:** 2024-03-13

**Authors:** Ross Holland, Guilherme Castro, Cecilia Chavana-Bryant, Ron Levy, Justin Moat, Thomas Robson, Tim Wilkinson, Phil Wilkes, Wanxin Yang, Mathias Disney

**Affiliations:** ^1^ East Point Geo, Ashgrove House, Monument Park, Chalgrove OX44 7RW, UK; ^2^ Department of Geography, University College London, Gower Street, London WC1E 6BT, UK; ^3^ Royal Botanic Gardens, Kew, Richmond TW9 3AE, UK; ^4^ Independent Researcher, Rayleigh SS6 9HB, UK; ^5^ Department of Geography, NERC NCEO, University College London, Gower Street, London WC1E 6BT, UK

**Keywords:** giant redwood, three-dimensional structure, carbon, lidar, biomass

## Abstract

Giant sequoias (*Sequoiadendron giganteum*) are some of the UK’s largest trees, despite only being introduced in the mid-nineteenth century. There are an estimated half a million giant sequoias and closely related coastal redwoods (*Sequoia sempervirens*) in the UK. Given the recent interest in planting more trees, partly due to their carbon sequestration potential and also their undoubted public appeal, an understanding of their growth capability is important. However, little is known about their growth and carbon uptake under UK conditions. Here, we focus on *S*. *giganteum* and use three-dimensional terrestrial laser scanning to perform detailed structural measurements of 97 individuals at three sites covering a range of different conditions, to estimate aboveground biomass (AGB) and annual biomass accumulation rates. We show that UK-grown *S. giganteum* can sequester carbon at a rate of 85 kg yr^−1^, varying with climate, management and age. We develop new UK-specific allometric models for *S. giganteum* that fit the observed AGB with *r* ^2^ > 0.93 and bias < 2% and can be used to estimate *S. giganteum* biomass more generally. This study provides the first estimate of the growth and carbon sequestration of UK open-grown *S. giganteum* and provides a baseline for estimating their longer-term carbon sequestration capacity.

## 1. Introduction

Large trees (>70 cm diameter at breast height, or DBH) are disproportionately important in terms of ecosystem service provision, particularly in terms of their carbon storage in aboveground biomass (AGB) [1–3]. *Sequoiadendron giganteum* (giant sequoia, also known colloquially in the UK as a giant redwood) grows rapidly, can live for over 3000 years and is the largest tree by volume in the world. *Sequoiadendron giganteum* groves in California also have the highest AGB of any ecosystem in the world [4], estimated to be up to 5800 Mg ha^−1^.

Recent work using non-destructive three-dimensional structural measurements performed either by specialized tree-climbing teams [5,6] or using terrestrial laser scanning (TLS) [7] has been used to estimate the AGB of individual *S. giganteum*. These studies show very close agreement with their respective estimates of AGB despite their different approaches. More generally, non-destructive methods for estimating tree mass and structure can help in decision-making about any species that is being considered for growth and carbon sequestration potential, particularly, when planted outside woodland or plantations.

### 1.1. Giant sequoia in the UK

*Sequoiadendron giganteum* seeds were first introduced to the UK in 1853 by the Scottish grain merchant Patrick Matthew. Later, in the same year, renowned nurseryman William Lobb returned with many more seeds and seedlings, along with accounts of the giant trees from which they came. Because of their rarity and novelty, these specimens commanded premium prices [8]. *Sequoiadendron giganteum* trees quickly became a symbol of wealth in Victorian Britain, where they were planted at the entrances of grand houses and estates, along avenues and within churchyards and parks.

The presence of *S. giganteum* in the UK is particularly interesting because the UK lacks the narrow adaptive niches of the species’ native range; however, they have seemingly thrived and are already some of the largest trees in the UK within 170 years after their introduction. Crucially, the planting dates of many of these trees are known due to the history of their introduction (*ibid*.). This means that we can estimate how fast they have grown and their rate of carbon sequestration. This is potentially important as there has been significant interest recently (since the 2000s) in planting both *S. giganteum* and *S. sempervirens* [9]. This is partly due to the surge in planting trees as a general reaction to the climate crisis, specifically for their potential to sequester carbon rapidly [10]. There have even been attempts to promote planting *S. giganteum* as a way to offset personal carbon footprints (https://onelifeonetree.com). Despite the highly questionable nature of this approach more generally [11–13], there is a lack of evidence to assess the benefits, i.e. how much carbon the UK-grown *S. giganteum* can sequester.

The apparent success of *S. giganteum* in the UK has also raised interest in their utility as commercial and amenity trees that may be resilient to the changing climate and environment, particularly rainfall, soil moisture and fire, as they are in their natural range [14,15]. The research arm of the UK’s Forestry Commission, Forest Research, has investigated the viability of *S. giganteum* as a climate- and disease-resilient species for diversifying UK commercial forestry [16]. Forestry England [17] estimates that there are already over half a million *S. giganteum* and *S. sempervirens* in the UK (and more are being planted). Just how resilient these trees are in the UK is an open question. Quantifying their carbon sequestration will help in making informed decisions about where (or even whether) to plant *S. giganteum* and how to manage them successfully.

**Figure 1.**
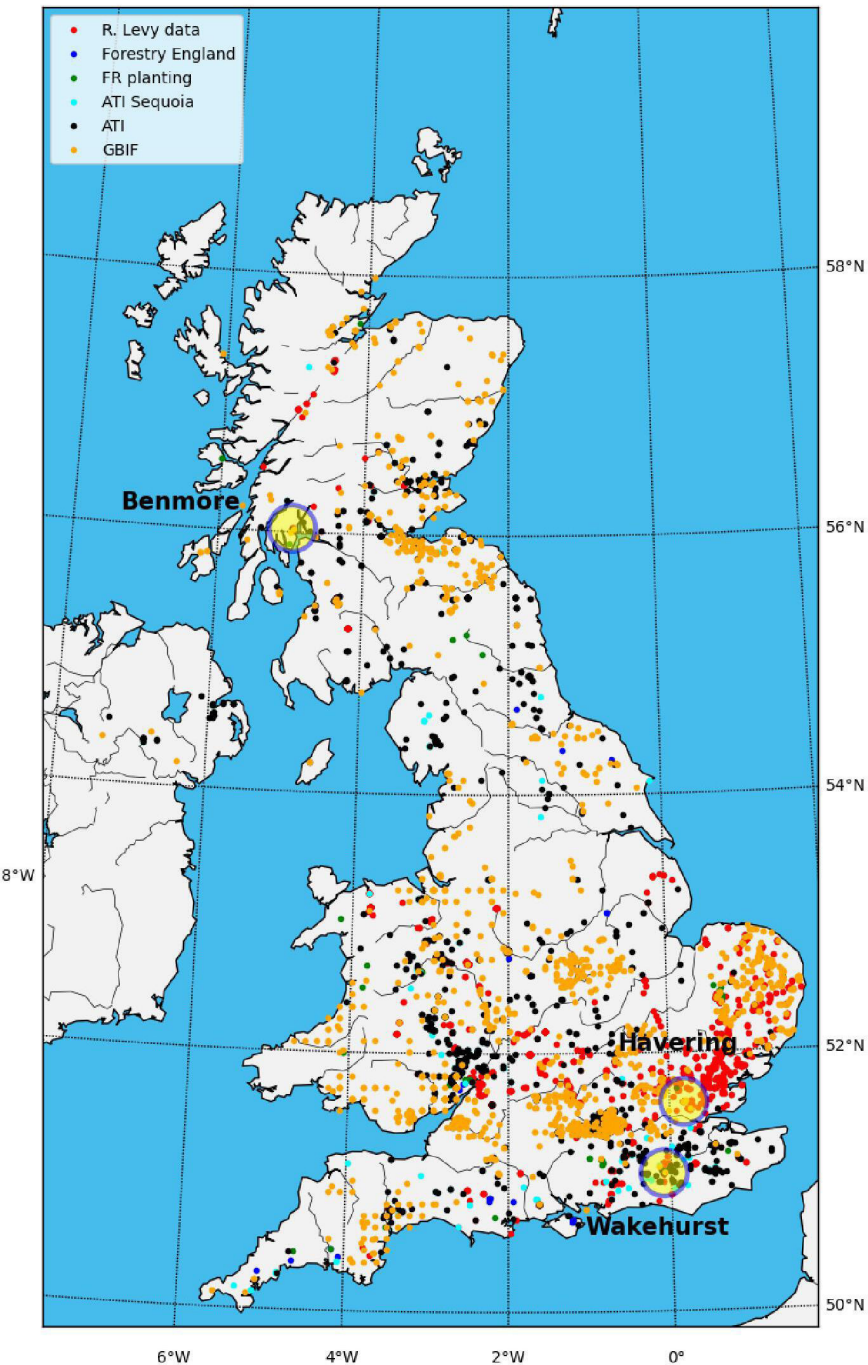
Locations of 4949 *S*. *giganteum* in the UK. Red points are 1821 trees from http://www.redwoodworld.co.uk/ (R. Levy, personal communication); 1505 trees are from the Woodland Trust Ancient Tree Index (ATI) [19] database; 24 trees are taken from the Forestry England ‘Route for Redwoods’ map (https://www.forestryengland.uk/route-for-redwoods); 39 locations are from the Forestry Commission planting trials (C. Reynolds, personal communication); 1560 trees are from the Global Biodiversity Information Facility [20]. The three sites used in this study are also marked.

Figure 1 shows a map of the location of 4949 *S*. *giganteum* identified from various sources across the UK. This map mostly only includes trees that are ‘notable’ in some respect (‘a tree which is significant locally, because it is special or particularly large compared with the trees around it. Notable trees are usually mature, but not always’ [18]). As a result, this map is far from comprehensive but is the only one we are aware of that identifies *S. giganteum* specifically. The data are skewed to South East England partly due to data sources. However, as many of the oldest and hence most notable trees were planted on the grounds of large houses and parks, this map may, in part, reflect the distribution of such sites in the UK in the mid-to-late nineteenth century but it might also reflect climate and terrain suitability.

In this study, we use new three-dimensional TLS measurements to estimate AGB and other structural attributes of *S. giganteum* at 3 locations in the UK. We use the known planting dates to estimate the approximate biomass accumulation rates and hence the carbon uptake of these trees. We assess how well the existing allometric models derived for *S. giganteum* in the USA perform on UK trees by comparing allometric and TLS-derived AGB. Finally, we develop new UK-specific allometric models for the UK-grown *S. giganteum* that can be used more widely to assess the carbon sequestration of these trees in the UK.

## 2. Materials and methods

### 2.1. Study sites

TLS data were collected at three different sites in April 2022 and April 2023. The site names and locations are given in table 1 and figure 1, along with meteorological conditions for the period 1991–2020. The three sites span a range of conditions, particularly with respect to rainfall. Essex (covering the Havering site) has the lowest mean annual rainfall in the UK over this period, while southeastern Scotland (covering Benmore) has the highest [21].

**Table 1.** Site details and meteorological characterization.

**site name**	**location (lat., long.), number of trees**	**mean T_min,max_ °C and sunshine hours**	**mean annual precipitation (mm)**
Benmore (botanical garden)	Dunoon, Argyll and Bute, Scotland (56.02 N, −4.98 W), 40 trees along a wide avenue	6, 12, with 1200 hours sunshine per year	1600
Kew Wakehurst Place (botanical garden)	Ardingly, West Sussex (51.06 N, −0.08 W), 7 well-spaced individuals close to the main house, and 16 sited in a woodland area, of two different age classes	6, 15, with 1600 hours sunshine per year	800
Havering Country Park (council-owned country park)	Havering-atte-Bower, Greater London (51.61 N, 0.17 E), 34 trees along a path through mixed woodland	6, 15, with 1600 hours sunshine per year	550

**Figure 2.**
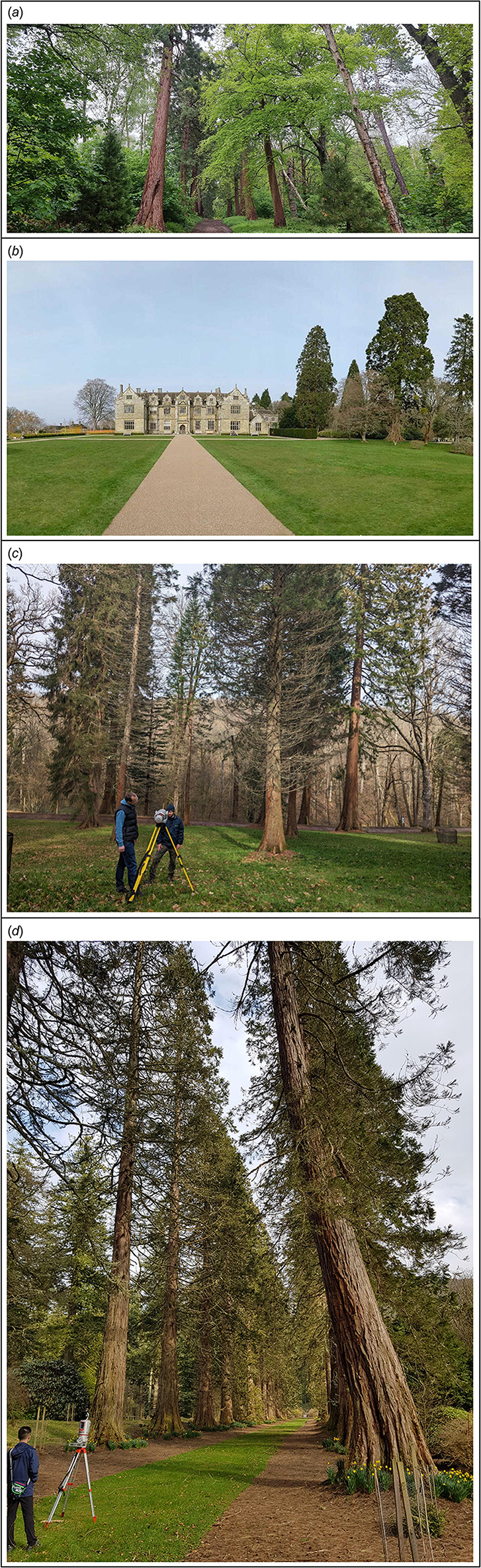
A general view of each of the three study sites where AGB of *S. giganteum* was estimated using TLS. (*a*) Havering Country Park. Image M. Disney. (*b*) Wakehurst Place: oldest trees next to the house. Image M. Disney. (*c*) Wakehurst Place: younger trees in the arboretum. Image M. Disney. (*d*) Benmore. Image: T. Wilkinson.

All tree samples were *S. giganteum*. Due to occlusions in some TLS point clouds, nine trees were omitted, leaving 97 trees in total. At Wakehurst, the average spacing between individual trees varies from ~17 m for the oldest trees close to the main house to ~10 m for the younger trees. At Benmore and Havering, the avenue form of the trees means that tree spacing is rather consistent at ~12 m and ~10 m, respectively. Benmore has only *S. giganteum* in the study area, Wakehurst has other species and a range of different sites with *S. giganteum*, whereas Havering is a mixed woodland, with *S. giganteum* on the edge of a path surrounded by other trees (see figure 2). Unlike Havering, the two botanical gardens at Benmore and Wakehurst are well-maintained to ensure optimal health of the plants, including work to aerate and decompact the soil around the oldest trees. At Wakehurst, protective fences have been placed around the largest and oldest *S. giganteum* where footfall is high to reduce soil compaction.

The oldest *S. giganteum* trees in this study are at Benmore and are some of the oldest trees in the UK. The avenue was planted in 1863 by the American owner of Benmore at that time, Piers Patrick [22]. The ages of *S. giganteum* at Wakehurst are less clear, given somewhat patchy historical records. The oldest were planted next to the main house between 1890 and 1910 (G. Costa, personal communication) with some having suffered top damage due to lightning over the years. In the wider Wakehurst area, *S. giganteum* trees are planted in a mixed arboretum/woodland environment, a few from 1910 to 1920, and then some in 1989 following the loss of other trees in the 1987 storm. The *S. giganteum* trees at Havering were planted around 1870 by the McIntosh family, who owned the Havering Palace. The palace was demolished in 1925 but there are still over 100 *S*. *giganteum* trees at Havering, making it the second-largest collection of them in the UK [23].

**Figure 3.**
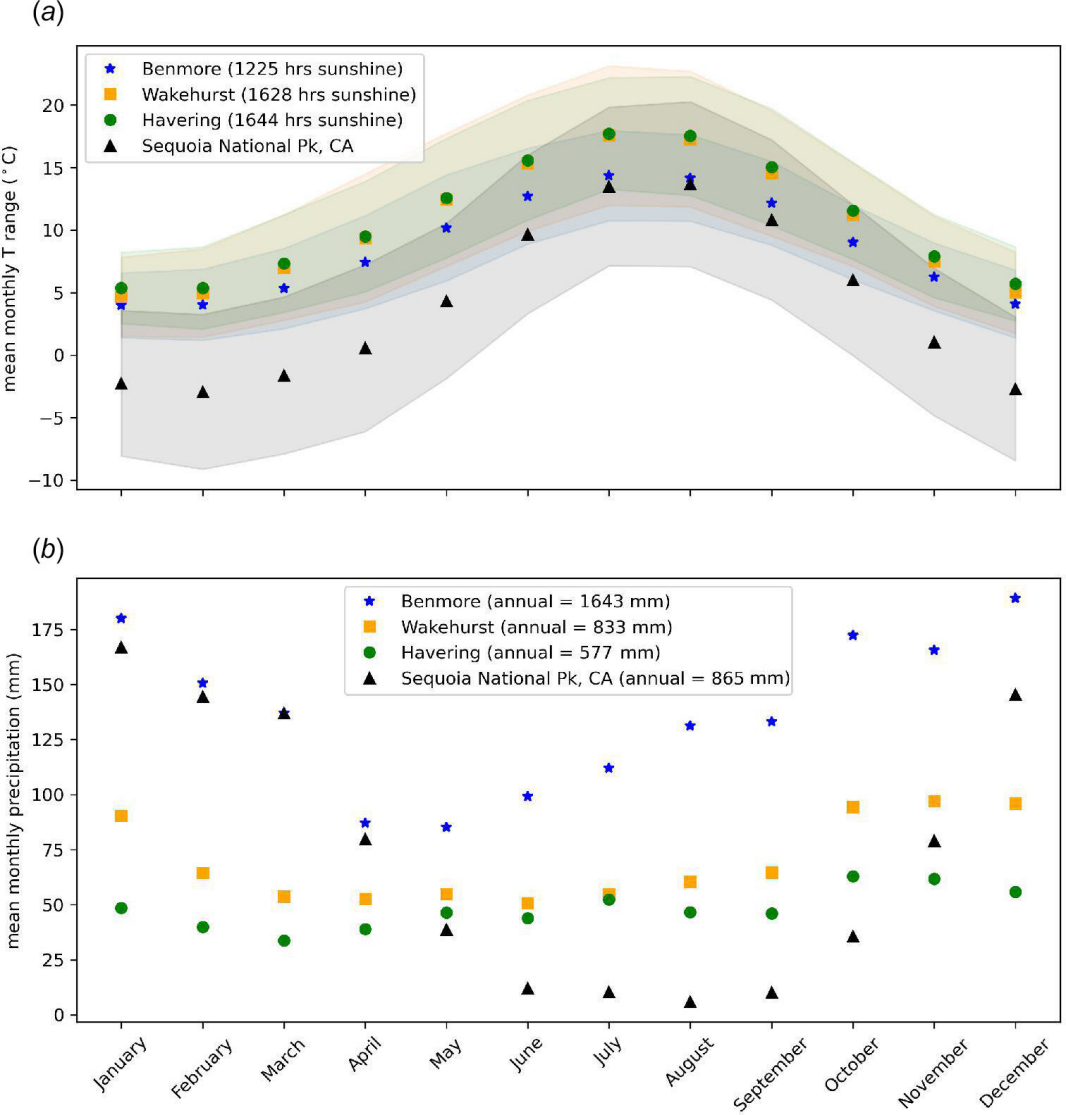
1991–2020 mean monthly T range (*a*) and rainfall (*b*) at all three UK sites (UK data from [21]; and Sequoia National Park, CA (36.48 N, −118.564 W), elevation 3034 m, data from PRISM [24]).

Figure 3 shows the mean monthly temperature, T, and precipitation across the three UK sites over 1991–2020. For comparison, data are shown for the same period from Sequoia National Park, CA, United States, a key *S. giganteum* site. Given the elevation (>3000 m), the latter site is far colder overall with significant winter snowfall, which can provide soil moisture in summer months even during periods with little or no rainfall. The summer temperatures at Sequoia National Park are particularly similar to those at Benmore. The annual mean precipitation is similar to that at Wakehurst but much more variable.

### 2.2. Terrestrial laser scanning measurements

#### 2.2.1. Data collection

TLS data were collected with *RIEGL* VZ-400i V-Line 3D TLS [25]. The scanner has a range of ~700 m and a beam divergence of 0.35 milliradians and emits light at 1550 nm. At each location, an upright scan and a scan at 90° tilt were taken. The angular resolution of each scan was 0.04°. The scanner was mounted on a tripod at 1.5 m above the ground. At Havering and Benmore, due to the avenue layout, scans were conducted ~10 m apart [26] down to the centre of the avenue and then at either side of each line of trees to try to ensure full coverage around all trees. At Wakehurst, the trees were sampled in a radial pattern, again at ~10 m apart, with the chosen locations.

#### 2.2.2. Data pre-processing

TLS scans from all positions were co-registered into point clouds for each plot using the Riegl RiSCAN PRO software (v 2.7.1). Individual tree point clouds were extracted manually from plot-level point clouds using the segmentation tool in CloudCompare (v 2.12.0).

#### 2.2.3. Estimating the structural properties of individual tree point clouds

Segmented point clouds of individual trees were extracted using the *TLSeparation* [27] and *TLS2trees* [28] Python tools. Tree volumes were calculated by fitting quantitative structural models (QSMs) to the resulting leaf-off point clouds using *TreeQSM* [29,30] and optimized using the *optqsm* tool [31]. This selects optimal parameters to build QSMs, runs the process 10 times for each tree and finds the mean volume and standard deviation (SD) of the 10 models. Along with the volumes, QSMs provide estimations of several other tree characteristics, including the height (H) (the distance between the lowest and the highest point of point clouds) and trunk and branch volume. The estimated volume of each tree, therefore, includes all woody materials, with needles removed as far as possible during the filtering process, representing the total tree volume, including barks.

#### 2.2.4. Aboveground biomass and growth rate estimation

AGB was estimated from the TLS data by multiplying TLS-derived (total over-bark, woody) volumes by a wood density value of 0.34 Mg m^−3^ [32]. Published values for the wood density of *S. giganteum* are in the range of 0.3 to 0.4 Mg m^−3^ [33] varying within and between individual trees. The TLS estimates of the volume assume no hollows within the trunk and do not differentiate wood or bark fraction. Sillett *et al*. [34] showed that *S. giganteum* bark fraction can be substantial and variable, up to 20% of the diameter in the lower trunk of younger trees, then reducing considerably with height and age. They also showed that the density of *S. giganteum* varies substantially, from 0.2 for bark, 0.26 for heartwood, and 0.34 for sapwood, up to 0.45 for branch wood. As we do not know the relative volumes of each component, we have used a value of 0.34 Mg m^−3^ either to convert from the volume to AGB or to scale AGB values for models that only work in AGB [33,35]. This will not affect the comparison of volumes within and between sites, only resulting in the AGB values.

DBH, defined as the diameter of the tree trunk at approximately 1.3 m above the ground [36], was calculated from the TLS point clouds by taking a cross-section of each tree point cloud between 1.2 and 1.4 m above the lowest point. The cross-section was flattened onto a single plane of *x* and *y* coordinates and the least squares circle [37, method_2b] was applied to estimate the diameter of a circle which best fits these coordinates [7]. Errors in DBH estimation result from fitting a circle to the (often) non-circular shape of a trunk. Diameter at the top of the buttress (DTB) is an alternative measure that is calculated as above, except that the cross-section is taken between 4.0 and 4.5 m above the lowest point to ensure that the cross-section is comfortably above any buttresses [5,7]. Another alternative ‘diameter’ measurement is the so-called ‘functional diameter at breast height’ (fDBH), defined as the diameter of a circle of an equivalent area to that of the cross-sectional area of the trunk at breast height [5]. fDBH was estimated here using the Python *alphashape* toolbox (v1.3.1) [38]. This involves fitting a convex hull to the DBH cross-section coordinates and then calculating the diameter of the resulting area if recast as a circle.

Annual growth rates for each site were calculated as follows:


$$Height\;growth\;rate=H_{mean}/age$$

$$DBH\;growth\;rate=DBH_{mean}/age$$

$$AGB\;accumulation\;rate=AGB_{mean}/age$$

These rates assume that the growth is constant across the full lifetime of a tree. In practice, growth rates tend to vary with age, particularly for much older trees [34]. In addition, by definition, we are only considering trees that have survived; we know of cases where trees have been replaced due to lightning strikes or had their top growth curtailed (e.g. at Benmore and Wakehurst), which is likely to affect the overall growth rates as assumed above. Lastly, converting AGB accumulation rates to carbon uptake requires the use of a carbon fraction (CF) coefficient. This is often assumed to be 0.5 [39]. However, the CF is known to vary with species, age, competition and management [40]. We have used CF = 0.54, as reported for *S. giganteum* by Lamlom & Savidge [41], who noted that this value is the highest of all the 41 North American tree species they measured. In practice, carbon accumulation is a function of various ecophysiological processes, particularly photosynthetic efficiency. Biomass accumulation estimated using allometric relationships is therefore widely used as an integrating proxy for these processes [42].

#### 2.2.5. Comparison of terrestrial laser scanning-derived aboveground biomass against allometric models

TLS-derived AGB was compared with AGB derived from five allometric models listed in table 2. Parks [43] calibrated a model based on H and DBH to estimate *S. giganteum* volume using samples of trunks and branches from fallen trees. Jenkins *et al*. [35] and Chojnacky *et al*. [33] developed generalized conifer allometric models, calibrated using cedar and larch trees, and also applied widely by the US Forestry Service (USFS) to estimate the AGB of both *S. giganteum* and *S. sempervirens* and Sillett *et al*. [5,6] developed several new allometric models for *S. giganteum*. These studies used either manual three-dimensional crown mapping [6] or ground-based/ladder-assisted methods [5] for estimating independent variables used to calibrate allometric models. In both cases, these models use DTB (rather than DBH) and crown volume (CV) to predict the tree volume. In this study, the CV was estimated using the measurements of the crown radius and crown depth (a vertical distance from the crown base to the highest leaf) and assuming the crown to be a conoid, paraboloid, or prolate spheroid depending on the observed shape. Here, we have estimated the CV from the TLS point clouds by fitting convex hulls as this most closely resembles the shape of crowns [5,6].

**Table 2.** The allometric models used in this study. V is the tree volume (m^3^); DTB is the diameter at the top of buttress (m); fDBH is the functional DBH (m); CV is the crown volume (m^3^).

**model**	**form**	***a***	***b***	***c***	***d***
Parks [43]	$log_{10}\left(V\right)=alog_{10}\left(DBH^{2}H\right)-b$	0.9246	0.4147	N/A	N/A
Chojnacky *et al*. [33]	$AGB=aDTB^{b}+cfDBH^{d}$	3.9656 x× 10^−4^	2.3122	1.9583 × 10^−3^	1.8657
Jenkins *et al*. [35]	ln(AGB)=a+bln(DBH)	−2.0336	2.2592	N/A	N/A
Sillett *et al*. [6]	$V=aDTB^{b}+cCV^{d}$	1.1588 × 10^−3^	2.1562	1.0898 × 10^−4^	1.4963
Sillett *et al*. [5]	$V=aDTB^{b}+cCV^{d}$	1.95 × 10^−3^	2.1	1.78 × 10^−4^	1.43

We have also developed four new allometric models of the UK *S. giganteum* volume, calibrated against the volume estimates from TLS and using DBH, H, DTB and CV as the dependent variables. These models were fitted using simple two parameter model forms given in table 2. All model fits were achieved using the function ‘*curve_fit*’ from the Python library *scipy.optimize* [44] using nonlinear least squares. This allows fitting in the arithmetic space, avoiding the need for log transformation and subsequent bias correction [45].

## 3. Results

### 3.1. Tree attributes retrieved from terrestrial laser scanning

The tallest of the 97 trees measured in this study was at Benmore with H = 54.87 m, volume = 36.21 ± 0.41 m^3^. This is not surprising given that Benmore is the oldest of the three groups of trees, and some of the oldest in the UK. By contrast, the shortest tree was at Wakehurst with H = 10.86 m, volume = 2.05 ± 0.03 m^3^. The tree with the largest volume was also at Benmore (B_T30), estimated at 59.92 ± 0.87 m^3^; however, it was only the seventh tallest tree with H = 49.99 m. The tree with the lowest estimated volume was one of the younger trees at Wakehurst (~33 years) 4.28 ± 0.09 m^3^, with H = 17.44 m and DBH = 0.32 m. The average height of trees at each site was estimated at 44.3 m in Benmore, 20.1 m in Wakehurst and 24.5 m in Havering. For Wakehurst, the younger cohort (13 trees aged <50 years) has a mean height of 22.8 m, while for the older trees (10 trees aged >100 years), the mean height is 35.0 m. The average volumes were 34.9 m^3^ in Benmore, 16.2 m^3^ in Wakehurst (4.2 m^3^ for the younger trees and 31.9 m^3^ for the older) and 11.8 m^3^ in Havering. The full dataset of TLS-derived tree data including the height, DBH, DTB, fDBH and volume can be found in the electronic supplementary material, tables S1–3.

**Figure 4.**
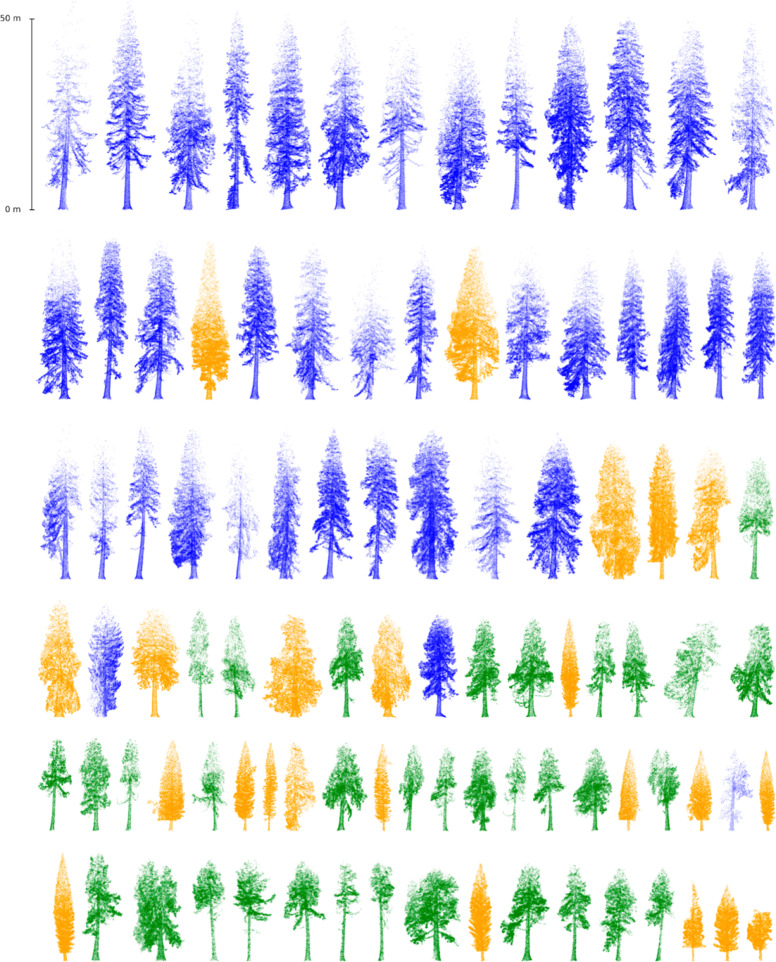
All trees measured in this study, ordered by height. Benmore (blue), Wakehurst (orange) and Havering (green).

Figure 4 shows a side-by-side comparison of the point clouds of all 97 individual trees, ordered by height. The trees at Benmore are nearly double the height of those at Havering, despite an age difference of only ~25 years. The trees at Wakehurst vary in age, with the oldest being ~50 years younger than at Benmore, and ~30 years younger than Havering, and the youngest being <40 years. This age difference leads to lower H but with generally fuller crowns for the older trees. Despite the greater height of the trees at Benmore, the average volume and thus biomass are similar to those of the largest Wakehurst trees (11.9 Mg versus 10.8 Mg, respectively).

**Figure 5.**
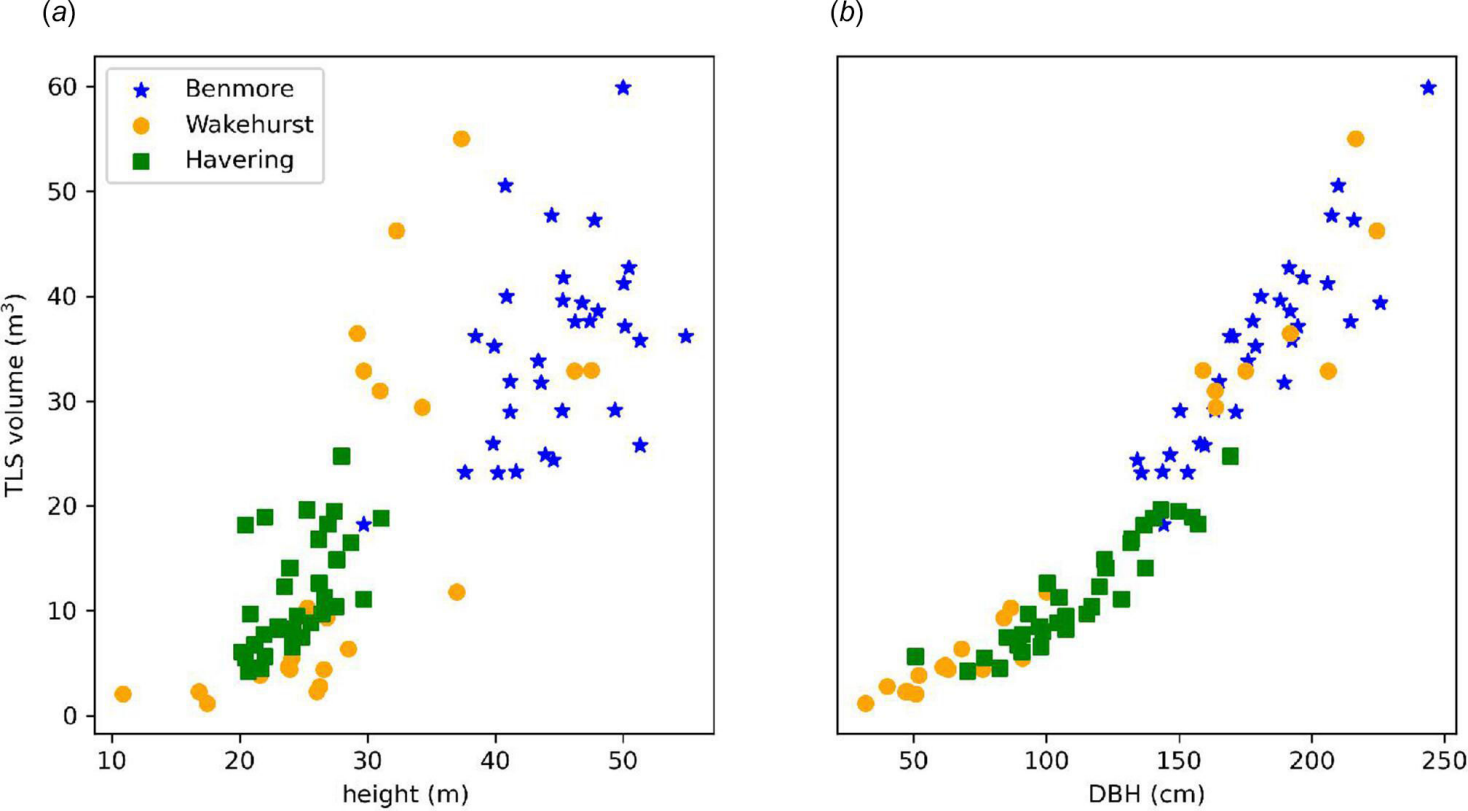
TLS-derived volume as a function of tree height (*a*) and DBH (*b*) across all sites.

Figure 5 shows the relationship between tree height and DBH with the TLS-derived volume of all trees and highlights the variability in the height and volume of trees across different sites. Three Wakehurst trees in this sample with a height of 35–40 m have volumes between 12 and 55 m^3^. This illustrates the difficulty of estimating AGB from height alone, e.g. using remotely sensed estimates of tree and canopy height. Figure 5 also shows a much stronger relationship between DBH and volume, albeit with greater variation with increasing DBH. At the upper end, a DBH of 220–230 cm corresponds to a volume between 35 and 55 m^3^. It is noticeable that the youngest trees at Wakehurst lie on the same trajectory in terms of DBH.

**Figure 6.**
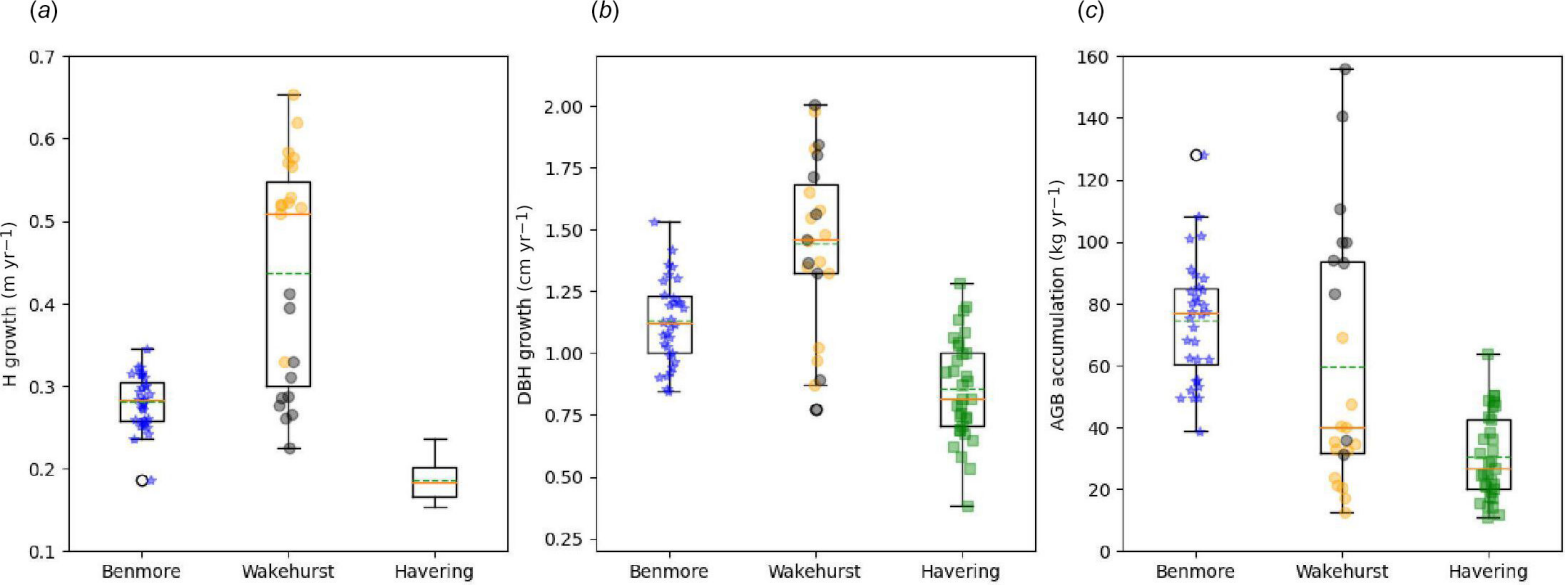
Box-whisker plots showing the mean annual increments of: height (*a*); DBH (*b*); and AGB (*c*) for all trees across the three UK sites. For Wakehurst, the trees are separated into two age classes: <50 years (orange) and ≥50 years (grey). Boxes show the quartiles Q1 to Q3, with the orange line at the median and the green dashed line at the mean. The upper whisker shows the last point <Q3 + 1.5 IQR and the lower, the last point >Q1 – 1.5 IQR, where IQR is the interquartile range (Q3–Q1). The locations of each individual tree on the *x*-axis are jittered randomly so that they are more easily visible. The Benmore and Wakehurst AGB accumulation rates are the only distributions that overlap statistically (*t*‐test *p* = 0.08, all other *p* < 10^−5^).

Given that the planting dates are known for all three sites, it is possible to estimate the mean annual growth rate of each tree, which is shown in figure 6.

Although taller, the Benmore trees have grown in height more slowly than those at Wakehurst at 0.28 m yr^−1^. The Wakehurst trees as a whole increased in height significantly faster at 0.44 m yr^−1^, but this is driven by the young trees. These have grown taller almost twice as fast (0.54 m yr^−1^) as the older trees (0.31 m yr^−1^), which have grown at a very similar rate to those at Benmore. The Havering trees grew much slower at 0.16 m yr^−1^. The growth rate of DBH follows the same pattern, with that of the Benmore trees increasing by 1.13 cm yr^−1^ on average, compared with 1.44 cm yr^−1^ at Wakehurst and 0.74 cm yr^−1^ at Havering, only just over half that of Wakehurst. For DBH, there is less of a split between the age cohorts at Wakehurst, with the older trees actually increasing in DBH slightly faster than the younger ones, at 1.47 and 1.42 m yr^−1^, respectively. In terms of AGB, Benmore has the highest mean accumulation rate at 74.7 kg yr^−1^, followed by Wakehurst at 59.6 kg yr^−1^ and then Havering at 30.4 kg yr^−1^. The variance at Wakehurst is much larger than that for the other two sites, reflecting the large variation in both H and DBH growth rates among the two age cohorts, with the mean being 32.9 kg yr^−1^ for the younger trees, and 94.4 kg yr^−1^ for the oldest ones. So although the mean annual AGB accumulation is lower than that at Benmore, some of the oldest trees are accumulating AGB at >100 kg yr^−1^.

### 3.2. Aboveground biomass estimates from allometric models and terrestrial laser scanning

**Figure 7.**
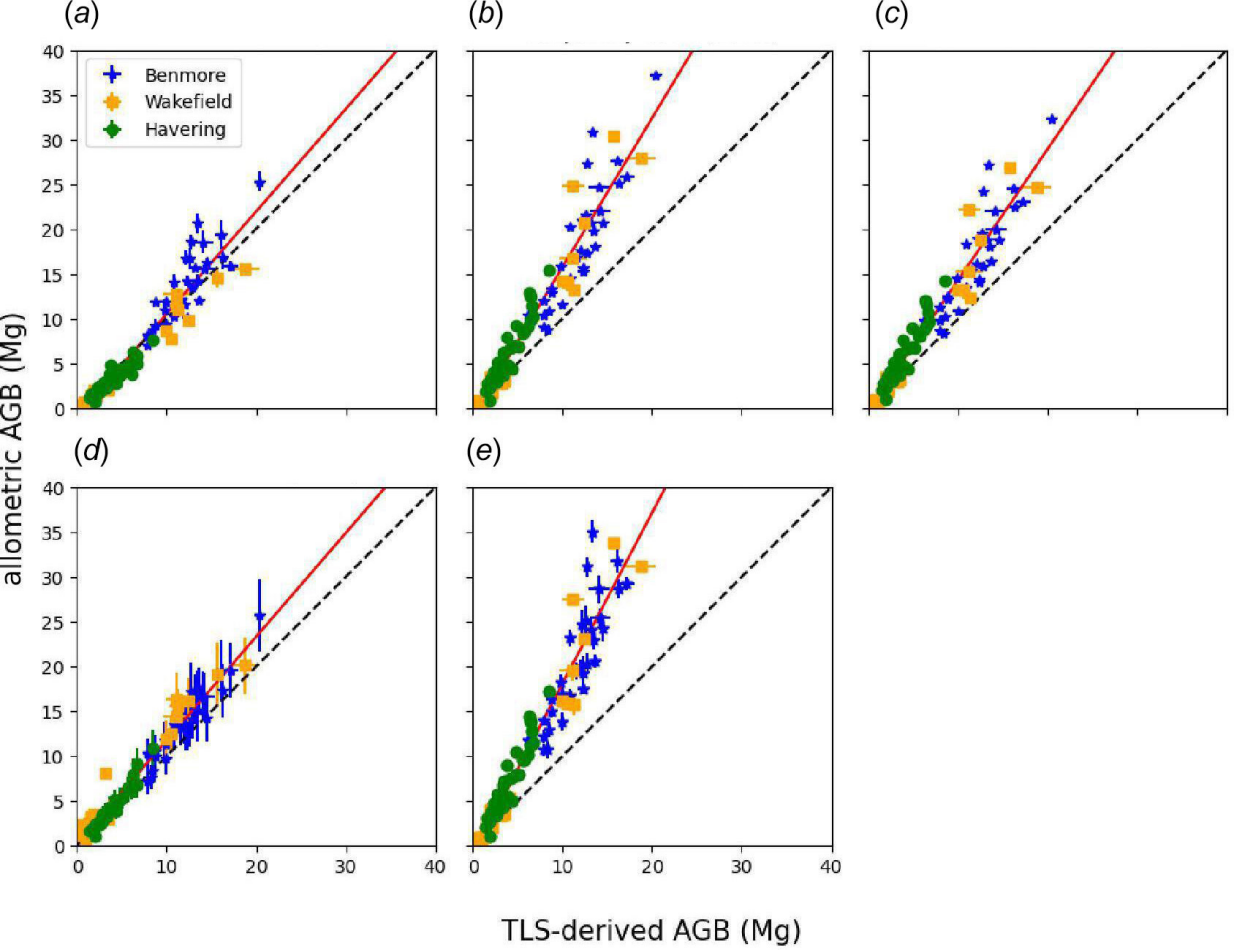
Comparison between TLS-derived AGB and allometrically derived AGB for all trees: (*a*) Parks [43]; (*b*) Chojnacky *et al.* [33]; (*c*) Jenkins *et al.* [35]; (*d*) Sillett *et al.* [6]; (*e*) Sillett *et al.* [5]. Volume is converted to AGB using the wood density value given in each study. Error bars for TLS-derived AGB represent the standard deviation of the mean of 10 QSM fits to each tree point cloud; error bars for the allometric estimates are derived using the standard deviation of the mean retrieved structural parameters for each tree, required for each model (i.e. DBH, fDBH, DTB, H and CV). The red line in each case is the fitted model; black dashed line is 1:1. Model parameters are shown in table 3.

Figure 7 shows a comparison between AGB estimates from the five published *S. giganteum* allometric models in table 2 and TLS-derived estimates.

**Table 3.** Comparisons between allometric and TLS-derived AGB shown in figure 7.

**model**	**gradient**	**intercept**	**RMSE (Mg)**	***r* ^2^**	**standard error**
Parks [43]	1.14	−0.95	1.69	0.92	0.04
Chojnacky *et al*. [33]	1.66	−0.90	2.37	0.92	0.05
Jenkins *et al*. [35]	1.46	−0.29	2.02	0.93	0.04
Sillett *et al*. [6]	1.16	0.18	1.16	0.96	0.03
Sillett *et al*. [5]	1.91	−0.96	2.56	0.93	0.06

### 3.3. New UK-specific allometric models for the UK *Sequoiadendron giganteum*

**Figure 8.**
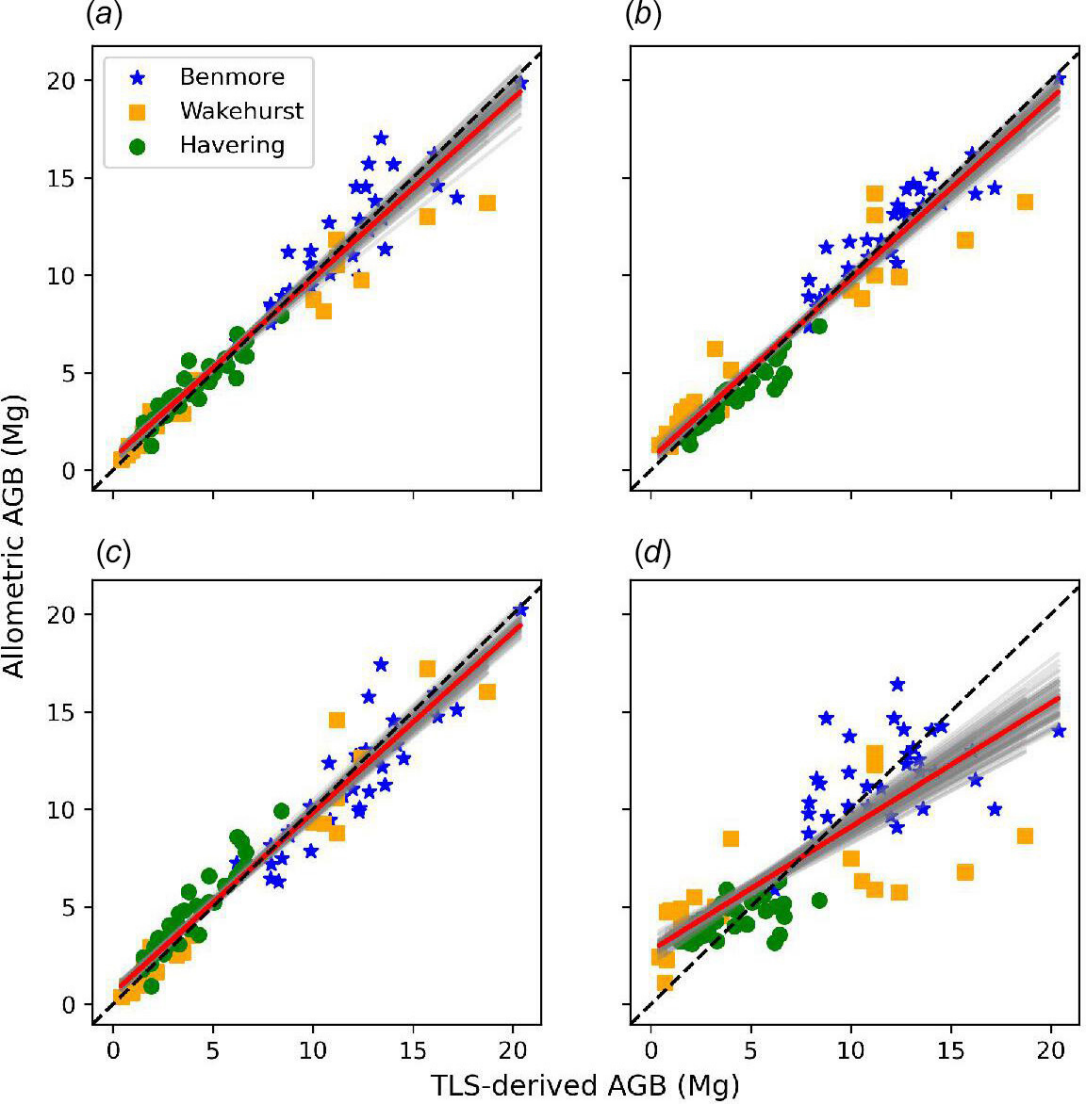
Comparison of the UK-grown *S. giganteum* AGB estimated using allometric models fitted to DBH, H and DTB derived from TLS data, and AGB estimated directly from TLS. (*a* and *b*) two variables (a(DBH^2^H)*^b^* and a(DTB^2^H)*^b^*); (*c* and *d*) single variable (aDBH*^b^* and aH*^b^*). The red line in each case is the fitted model; black dashed line is 1:1. Grey regions are parametric bootstrap fits, across 100 samples in each case.

Four new allometric models were developed for estimating the volume (and AGB) of the UK-grown *S. giganteum*. The forms of these models and the regression parameters derived from fitting to the TLS-derived estimates of H, DBH and DTB are given in table 4. The comparison between AGB estimates derived from TLS and AGB predicted by the four new UK-specific *S. giganteum* allometric models is shown in figure 8.

**Table 4.** New allometric models for estimating the UK-grown *S. giganteum* volume—model form, parameters and model fit properties in each case. H, DBH and DBH are as defined above.

**model**	**form**	***a***	***b***	***m***	***c***	**RMSE (Mg)**	***r* ^2^**
DBH and H	*a*(*DBH^2^H*)*^b^*	0.374	0.697	1.016	−0.168	1.261	0.937
DTB and H	*a*(*DTB^2^H*)*^b^*	0.423	0.750	1.011	−0.114	1.298	0.933
DBH only	*aDBH^b^*	3.542	1.956	1.009	−0.099	1.282	0.935
H only	*aH^b^*	0.021	1.663	1.049	−0.458	2.897	0.665

The results in figure 8 and table 4 show that three of the four new models can predict AGB with *r* ^2^ > 0.93, bias < 2% and RMSE of <1.3 Mg. The H-only model is the poorest fit of the four, but even in this case (the most appropriate for ALS-derived estimations of AGB), the model has *r* ^2^ > 0.67 and bias < 5%. Both the two-variable models using either DBH or DTB, and H are almost identical. The DTB version is included as DTB is required when trees have significant buttresses at 1.3 m where DBH would otherwise be biased by irregular trunk shapes. The DBH-only model is the second best-performing model in terms of *r* ^2^ and RMSE and the best in terms of bias.

## 4. Discussion

In this study, we used TLS to estimate the volume, biomass and biomass accumulation rates of *S. giganteum* in three sites across the UK. We demonstrate that when planting dates are known, TLS can be used to estimate the biomass uptake of *S. giganteum* non-destructively and develop allometric size-to-mass models. As noted above, TLS estimates the total wood (over bark) volume, excluding foliage, so a single value of wood density representing trunk or branch wood will result in a slight overestimate of AGB, all else being equal.

The approach we demonstrate in this study is more generally applicable for estimating the carbon accumulation rates of planted trees, particularly when information is unavailable on local growth rates (e.g. for introduced species) and/or for trees outside woodlands that may be planted for a variety of reasons other than for commercial exploitation.

### 4.1. Environmental factors leading to different growth rates

The growth rate of *S. giganteum* varied between the three study sites, as well as with age at a single site, Wakehurst Place. Intra-site variation in the tree size and form is evident but is even more striking across the different sites (figure 4). The trees at Wakehurst have increased in height at the fastest rate, albeit varying strongly with age (younger trees increasing much more rapidly), followed by Benmore and Havering much more slowly. These patterns are repeated with DBH but with much greater within-site variance across all sites and a reversal of the age differences at Wakehurst, the older trees add more DBH per year than the younger ones. As a result, the Benmore and Wakehurst trees have accumulated AGB at similar rates but in slightly different ways: the greater increase with age at Wakehurst is driven by the DBH growth rate more than H, and with much greater variance overall. This also suggests that at Wakehurst at least, there is a gradual shift in form with age as the increase in height slows down, while DBH growth increases.

Various factors are likely to have influenced these differences in growth rates, particularly the relationship between DBH and H, including climate, soil, spacing/competition, disturbance and management. Water availability is known to have a particularly strong influence on the growth of *S. giganteum*, with drier years correlating with lower tree ring growth [46]. In the native range of *S. giganteum*, snow melt maintains high soil moisture during the drier summer months despite very low rainfall [47]. Benmore experiences the highest precipitation with consistently wetter months than either Wakehurst or Havering. Precipitation at Wakehurst is lower than that at Benmore, but average annual totals are still within the range seen for the US *S. giganteum* groves [5]. Havering has much lower average annual precipitation (35% of that at Benmore and 70% of that at Wakehurst), largely due to its dry winter months, caused by the ‘rain-shadow’ effect that shields eastern England from winter Atlantic depressions [48]. This is potentially one reason for the limited growth seen in the trees at Havering. In terms of temperature, Wakehurst and Havering have the same mean maximum and minimum temperatures while Benmore is cooler overall. Endemic groves experience high seasonal variability, with temperatures regularly at or below 0°C and snow cover in the winter, and then temperatures in the mid-20 °C in the summer [5,49]. Temperature is both more stable and less extreme in the UK sites. Given that all the sites fall well within the native temperature ranges, it is less likely that temperature will affect the growth of *S. giganteum* in the UK than rainfall. Based on this evidence, when deciding where to plant *S. giganteum* in the UK, it will be important to consider whether there is sufficient rainfall and year-round soil moisture, at the moment of planting and for the coming decades [50].

The physical and chemical properties of soils are another consideration. *Sequoiadendron giganteum* trees grow best in deep sandy loams and soil pH between 5.5 and 7.5 [49]. In all three locations in our study, the soil is categorized as sand to loamy sand, with a pH of ~6 and depth defined as intermediate/deep (can be easily dug to a depth of 1 m or more), therefore suitable for the species [51]. Both Horst-Heinen *et al*. [52] and Son & Chung [53] suggest that the soil depth exhibits at least some control over the height of conifer trees. Weatherspoon [49] highlights that *S. giganteum* trees have grown well in a diverse range of soils when planted in regions outside the endemic groves.

The height, DBH and allocation of branch mass of *S. giganteum* are all sensitive to the growing space [54]. *Sequoiadendron giganteum* is known to vary allocation strategies in response to competition (as well as with age), e.g. allocating more biomass to branch materials than to trunk materials with reduced light [55]. York *et al*. [56] found that DBH was more sensitive to growing space than height, with a sharp reduction in the DBH growth rate where forest stands are more dense. The trees at Wakehurst are the most widely spaced, followed by Benmore, with Havering trees being closest together. This may explain the growth differences in Wakehurst trees potentially experiencing lower competition compared to the other two sites. In addition to environmental factors, the growth response of the UK giant sequoia is directly affected by management actions. Conversely, fire is the main factor affecting competition in the native range of the species [6,15]. The grand house to which Havering avenue led was demolished in 1925 [57], with the land becoming the property of the local council, which has allowed native woodland to grow up around the giant sequoias (see figure 2). By contrast, Benmore and Wakehurst have continually remained part of botanic gardens, whose maintenance is intensive and focused on individual trees. The resulting AGB growth in younger individuals is dependent on interactions with surrounding trees, which change over time [34]. The more rapid increase in H than DBH at Benmore is consistent with studies showing that trees will grow tall, rather than wide when the spacing between trees is reduced [10,56,58].

### 4.2. The UK versus USA *Sequoiadendron giganteum*

Sillett *et al*. [6] found that *S. giganteum* in the USA of a similar age to those in the UK accumulate AGB at anywhere between ~30 and ~150 kg yr^−1^ (16.2–81 kg carbon yr^−1^) with accumulation increasing until their death, albeit not uniformly and likely declining as they exceed 500 years. In this study, the fastest growing *S. giganteum* in terms of AGB accumulation reached 156 kg yr^−1^ (84.2 kg carbon yr^−1^). The trees of Wakehurst and Benmore fall within the range of AGB accumulation found by Sillett *et al*. [6,59]. The trees at Havering average 26 kg yr^−1^ (14 kg carbon yr^−1^) falling well behind the other sites and just less than the slowest growing trees in Sillett *et al*. [6], again indicating that the growth of these trees has been restricted by one or more factors at that location.

Forests comprising *S. giganteum* and *S. sempervirens* are likely to be the most effective forests worldwide for carbon sequestration as they accumulate AGB at a faster rate than any other vegetation [42,59], maintain high rates of productivity throughout their potentially very long lives and are resistant to disturbance such as fire. This is also augmented by their particularly high carbon fraction [41]. Given that carbon sequestration is already being used as a justification for planting *S. giganteum* in the UK, our estimates can help inform that process. However, like many others, we note that focusing on trees primarily as carbon sinks, particularly when planted individually or in urban areas, risks overlooking their many other potentially more important benefits.

### 4.3. Performance of different allometric models in estimating aboveground biomass

The estimates of AGB from allometric models for *S. giganteum* and generic conifers (including giant sequoia) developed for US trees were compared with the TLS estimates of *S. giganteum* in the UK. For all models, 90% or more of the variation in estimated AGB can be explained by the independent variables. However, there was significant bias (over-estimation) using the US-derived allometric models, between 16% and 79% over all models. This is perhaps not surprising considering that these models were all calibrated against US-grown *S. giganteum*, generally of a much greater age than those measured here. The closest models to the TLS-derived AGB are Parks [43] in terms of bias (14%) and Sillett *et al*. [6] in terms of RMSE (1.16 Mg). This suggests that the form of the models is suitable, but that the US-grown trees are heavier for the equivalent DBH and H, perhaps due to much greater age and/or different growth conditions. The biomass accumulation rates of *S. giganteum* have been shown to increase with age [6], so using a model calibrated against large, old trees may well show this kind of bias when used for very much younger ones. This bias has been noted more generally as an issue when using allometric models to predict far out of their calibration sample [60]. Age and competition further affect the relative allocation of mass between the trunk and branch of *S. giganteum* and consequently will change the wood density (and carbon fraction). In general, the larger the DBH, the greater the variation we show between model predictions. This also suggests that the form of these allometric models is not size/age invariant as is often assumed [61]. For instance, Lutz *et al*. [62] noted the impact of the lack of large trees in allometric models specifically applied to *S. giganteum*.

Four new allometric models were developed, calibrated against the TLS-derived estimates of volume and AGB from *S. giganteum* trees from the UK, either using two variables (DBH or DTB and H), or a single variable (DBH and H, respectively). DBH and DTB can be measured manually; H can be estimated manually or measured from airborne or UAV lidar. DBH, DTB and H can also all be measured from TLS [63]. For both two-variable models, *r* ^2^ values were > 0.93 and bias < 2%. For the single-variable models, DBH was the strongest predictor of AGB and was almost as good as the two-variable DBH, H model. The weakest predictor was H alone, with *r* ^2^ = 0.64 and with bias < 5%. Considering the principle of parsimony and ease of measurement, the DBH-only model ought to be favoured [64]. This is encouraging as it suggests that DBH alone is an accurate predictor of AGB for the UK-grown *S. giganteum*, at least within the size and age ranges measured here. The H-only model might allow estimates of AGB from Earth observation (EO) data, specifically airborne, UAV or spaceborne lidar. The consistency of allometric fit seen across management and climatic differences suggests that these new models ought to be applicable across the UK more generally. As for all allometric models, if they are used in different conditions, or applied to trees of very different age and size from those used in calibration, this will result in much greater uncertainty in AGB prediction [60]. However, a major benefit of the TLS-based approach is that additional measurements can be easily made to recalibrate model fits, or even to develop site-specific models.

## 5. Conclusion

There is increased interest in planting giant sequoias in the UK, given their potential carbon sequestration capacity and apparent suitability to the UK climate. However, there is little quantitative evidence to support these observations. We used the TLS-derived estimates of tree volume and structure parameters (tree diameter and height) to estimate AGB, growth and carbon sequestration rates of *S. giganteum* at three sites in the UK spanning a range of age, growing and climate conditions. At two of the three sites sampled in this study (Wakehurst and Benmore), the trees seem to have at least matched average growth rates, in terms of AGB and carbon accumulation, of their counterparts of a similar age in the USA, at up to 150 kg AGB yr^−1^ (81 kg carbon yr^−1^). The trees at Havering have grown much more slowly (30 kg AGB yr^−1^, 16.2 kg carbon yr^−1^), likely limited by lower rainfall, increased competition and compounded by the absence of intensive management that has been applied at Benmore and Wakehurst. Given the growth of *S. giganteum* in the UK, particularly in open-grown well-managed conditions, they are likely to be a good choice purely from the perspective of carbon uptake. But like all planted trees, considerations of ecological impact, climate resilience, public perception, etc., are likely to be equally important.

We used allometric models calibrated on native US *S. giganteum* to predict the AGB of UK trees. These models typically explain the majority of observed variance (high correlation) but overestimate the AGB of UK trees compared to TLS-derived estimates. This is likely because they were calibrated using much older and larger trees than the ones present in the UK and across different climates. The US-based species-specific *S. giganteum* models also include some of the largest trees on the Earth and large specimens have been shown to skew allometric models. Four new UK-specific allometric models were developed, calibrated against the TLS-derived AGB using DBH or DTB and H, or DBH and H only. All of these were able to predict the AGB of TLS-derived estimates with an RMSE of <3%, equally well across all sites, with the DBH-only model being particularly effective. This work underscores the importance of developing locally calibrated models to estimate and predict carbon uptake accurately in *S. giganteum*, but also more generally for planted trees. The TLS-based approach can be used to test the existing allometric models, or to develop new species- or context-specific models and thus provide an important way to help understand and manage our planted trees.

## Data Availability

Individual TLS point clouds for extracted trees are deposited with Dryad [65]. All structural information derived from these point clouds that is the basis for the analysis above is included in the electronic supplementary material [66].
